# Current Issues in the Development of Foetal Growth References and Standards

**DOI:** 10.1007/s40471-018-0168-6

**Published:** 2018-09-20

**Authors:** Eric O. Ohuma, Tsi Njim, Megan C. Sharps

**Affiliations:** 1 Centre for Tropical Medicine and Global Health, Nuffield Department of Medicine University of Oxford, Old Road Campus, Oxford, OX3 7BN UK; 2 Liverpool School of Tropical Medicine, Pembroke Place, Liverpool, L3 5QA UK; 3 Maternal and Fetal Health Research Centre, Division of Developmental Biology and Medicine, School of Medical Sciences, Faculty of Biology, Medicine and Health, University of Manchester, Manchester, M13 9WL UK

**Keywords:** Foetal growth, References, Standards, Charts, Fetal measurements, Fetal biometry

## Abstract

**Purpose of Review:**

This paper discusses the current issues in the development of foetal charts and is informed by a scoping review of studies constructing charts between 2012 and 2018.

**Recent Findings:**

The scoping review of 20 articles revealed that there is still a lack of consensus on how foetal charts should be constructed and whether an international chart that can be applied across populations is feasible. Many of these charts are in clinical use today and directly affect the identification of at risk newborns that require treatment and nutritional strategies. However, there is no agreement on important design features such as inclusion and exclusion criteria; sample size and agreement on definitions such as what constitutes a healthy population of pregnant women that can be used for constructing foetal standards.

**Summary:**

This paper therefore reiterates some of these current issues and the scoping review showcases the heterogeneity in the studies developing foetal charts between 2012 and 2018. There is no consensus on these pertinent issues and hence if not resolved will lead to continued surge of foetal reference and standard charts which will only exacerbate the current problem of not being able to make direct comparisons of foetal size and growth across populations.

## Introduction

A reference or standard chart depicts a family of curves representing a few selected centiles of the distribution of some physical characteristic of the reference population as a function of age. Such charts allow an individual to be placed in the context of like individuals. Charts of measurements are useful for assessing humans at all stages: foetuses, neonates, children and adults. Adolphe Quetelet (1796–1874) was the first to investigate the statistical properties of anthropometry and apply the concept of the normal distribution to anthropometry data [1]. Francis Galton (1822–1911) introduced the use of percentile scores for comparing measurements with the normal distribution using data on attained height from birth to adulthood [2]. A first application of this approach was in growth in height, which is normally distributed from birth to adulthood conditional on age.

Foetal growth monitoring during pregnancy has been an important practice amongst obstetricians usually done to ascertain the health status of a foetus and relevant interventions may be provided when the health of a foetus is compromised [3, 4]. Growth charts are intended to aid clinical judgements. Foetal growth charts are primarily used: to compare the size of a foetus with reference data when gestational age (GA) is known at a specified time [5••]; to estimate GA from foetal size (e.g., crown-rump length and foetal head circumference are commonly used for this purpose) [6–8]; and to assess a foetus’ rate of growth between two time points (velocity) [9, 10]. For example, a foetus classified as being > 97th centile according to an estimated foetal weight chart would prompt clinicians to either deliver early or consider a caesarean section to avoid complications that may be associated with delivering a large baby.

The systematic review of 83 published reference charts of foetal biometry across 32 countries identified in 2012 by Ioannou et al. revealed wide variations between the centile values reported by published studies. There was considerable methodological heterogeneity: the charts were based on different populations and created with different sample selection, methodology and statistical modelling methods [11]. The availability of many charts in use is problematic as it has been shown that the choice of a reference chart in a particular setting could have great impact on the assessment of foetal biometric assessments [3, 12]. For example, a study by Salomon et al. evaluated the impact of using different charts and reported between 2.6 and 23.6% of measurements would be classified as abnormal using three different charts of foetal biometry that are commonly used [12]. Due to these differences in the data used in the creation of each foetal growth chart, comparisons between them are difficult.

These differences in foetal growth charts with the need to be able to make direct comparisons were the motivation for the World Health Organisation (WHO) in 1995 to advocate for the creation of a single universal chart that could be used globally to assess foetal and child growth which reflects recent health and feeding recommendations of different populations and settings [13]. This recommendation attracted several debates over the varying effects of various factors like environmental and genetic influences on foetal growth [14].

However, regardless of which growth chart is used clinically, there are design and methodological constructs that must be taken into consideration. To understand and summarise current issues in the developments of foetal references and standards, we did a quick review (by no means exhaustive) of studies published between 2012 and 2018 whose aim was to construct foetal references or standards. The review was aimed at understanding and highlighting the current debates and schools of thought regarding the development of foetal growth references and standards. We focussed on studies in the last 5 years as they represent a time period where three large prospective studies purposely designed to construct foetal growth charts for wide use were published i.e., the international foetal charts from the INTERGROWTH-21st Project [5••], the WHO foetal growth charts intended for international application [32••] and the National Institute of Child Health and Human Development foetal charts that were ethnic specific [15••].

## Scoping Review

### Search Strategy

We searched MEDLINE via PubMed from January 2012 to April 2018 to identify studies that constructed foetal growth charts for foetal growth assessment. The search strategy used was adapted from a previous systematic review by Ioannou et al. [11] shown in Table 1. In addition, reference lists of all articles included were similarly searched for potential relevant articles. The search included book chapters and documents from organisations such as WHO and the United States Centers for Disease Control and Prevention (CDC) but excluded abstracts from conference proceedings. The initial search yielded 180 articles. After a review of the titles and abstracts, 27 articles were selected for a closer consideration. From the 27 articles, 20 studies produced foetal growth references or standards. A summary of the characteristics of the included studies are shown in Table 2 with details on study designs used and the intended purpose of the study.

**Table 1:** Search strategy [11]

Search strategy	
(((fetal OR foetal OR fetus OR foetus) AND growth) OR ((fetal OR foetal OR fetus OR foetus) AND biometr*)) OR (reference adj (curve* OR chart* OR index OR indices OR equation* OR value* OR range* OR equation* OR centile* OR percentile*)) OR (biometr* adj (curve* OR chart* OR index OR indices OR equation* OR value* OR range* OR equation* OR centile* OR percentile*)) OR (size adj (chart* OR curve*)) OR (dating adj (curve* OR chart*))*Reference Values/Ultrasonography, Prenatal/(ultrasound* OR ultrasonogra* OR sonogra*)

**Table 2:** Summary of the characteristics of the included studies

Reference	Country	Sample size (number recruited)	Design	Type of population	Type of sampling	Recruitment period	Chorionicity	Foetal measurements	Statistical methodology	Type of chart	Intended use
Shivkumar et al. 2015 [16]	Canada	642	Retrospective	All inclusive	Hospital based	> 34 weeks	Twins	Weight	Multilevel linear regression models	Reference	Not clear
Daniel-Spiegel et al. 2016 [17]	Israel	79,328	Retrospective	All inclusive	Hospital based	14–42 weeks	Singleton	BPD, HC, AC and FL	Quintile regression	Reference	Local
Dias et al. 2016 [18]	Sri Lanka	4256	Cross-sectional	Healthy pop	Population based	11–13 weeks	Singleton	SFH	Linear and polynomial regression models	Not clear	Not clear
Xu et al. 2015 [19]	Ethnic Chinese	313	Longitudinal	Healthy pop	Hospital based	11–12 weeks	Singleton	HC, AC, FDL	Mixed-effect linear regression models	Standard	Local
Briceño et al. 2013 [3]	Colombia	792	Cross-sectional	Healthy pop	Hospital based	12–40 weeks	Singleton	HC, AC, FDL	Fractional polynomial regression models	Reference	Local
Liao et al. 2012 [20]	Brazil	200	Longitudinal	Healthy pop	Hospital based	< 21 weeks	Twins	HC, AC, FDL	Multilevel regression analysis	Reference	Local
Gabbay-Benziv et al. 2017 [21]	USA	2161	Retrospective	All inclusive	Hospital based	< 20 weeks	Twins	FL, BPD, HC	Linear mixed model	Reference	Local
Araujo Júnior et al. 2014 [22]	Brazil	31,476	Cross-sectional, retrospective	Healthy pop	Hospital based	< 14 weeks	Singleton	BPD, HC, AC, FL, EFW	Polynomial regression models	Reference	Local
Araujo Júnior et al. 2014 [23]	Brazil	333	Cross-sectional, retrospective	Not clear	Hospital based	14–38 weeks	Twin	BPD, AC, FL and EFW	Polynomial regression models	Reference	Local
Kwon et al. 2014 [24]	Korea	986	Cross-sectional	Healthy pop	Hospital based	15–19 weeks	Singleton	BPD, HC, AC, FL, TL, HL, UL	Fractional polynomial regression models	Reference	Local
Buck Louis et al. 2015 [15••]	USA	2334	Longitudinal	Healthy pop	Hospital based	8–13 weeks	Singleton	BPD, HC, AC, FL, HL	Linear mixed models with cubic splines	Standards	International/ethnic-specific
Pay et al. 2017 [25]	Norway	42,018	Retrospective	All inclusive	Hospital based	All gestational ages	Singleton	SFH	Non-linear regression of symphsio-fundal height	Reference	Local
Deter et al. 2016 [26]	USA	119	Longitudinal	Not clear	Hospital based	> 17 weeks	Singleton	BPD, HC, FDL, AC, ThC, HDL, MAC, FAV, FTV	Two-level mixed-effects modelling	Reference	Local
Jiang et al. 2013 [27]	Ethnic Chinese	6832	Cross-sectional	Healthy pop	Hospital based	> 16 weeks	Singleton	BPD, AC, FL	Polynomial regression models	Reference	Local
Sotiriadis et al. 2016 [28]	Ethnic Greek	1200	Longitudinal	All inclusive	Hospital based	> 16 weeks	Singleton	BPD, HC, AC, FL, FTV	Polynomial regression	Reference	Local
Stirrup et al. 2015 [29]	England	2025	Retrospective	All inclusive	Hospital based	> 14 weeks	Twin	BPD, HC, AC, FL	Multilevel mixed-effects statistical models	Reference	Local
Rizzo et al. 2016 [30]	Italy	8070	Retrospective	All inclusive	Hospital based	First trimester	Singleton	BPD, HC, AC, FL	Quantile regression	Reference	Local/gender-specific
Moety et al. 2015 [31]	Egypt	334	Cross-sectional	Healthy pop	Hospital based	20–41 weeks	Singleton	FTV	Multivariable linear regression model	Reference	Local
Papageorghiou et al. 2014 [5••]	International	13,108	Longitudinal	Healthy pop	Population based	9–14 weeks	Singleton	HC, BPD, AC, FL, OFD	Second-degree fractional polynomial modelling	Standards	International
Kiserud et al. 2017 [32]	International	1387	Longitudinal	Healthy pop	Hospital based	First trimester	Singleton	HC, BPD, AC, FL, HL, FFL	Polynomial regression methods, using the generalised estimating equations method	Standards	International

*BPD* foetal biparietal diameter, *HC* head circumference, *AC* abdominal circumference, *FL* femur length, *SFH* symphysis-pubis fundal height, *FDL* femur diaphysis length, *EFL* estimated foetal weight, *HL* humeral length, *UL* ulnar length, *ThC* mid-thigh circumference, *HDL* humerus diaphysis length, *MAC* mid-arm circumference, *FAV* fractional arm volume, *FTV* fractional thigh volume, *OFD* occipitofrontal diameter, *TSST* triceps and subscapular skinfold thicknesses, *FFL* foetal foot length, *NC* not clear

### Summary Findings from the Scoping Review

Of the 20 studies, three aimed to develop international foetal standards and were all longitudinal studies (the INTERGROWTH-21st Project, the NICHD Fetal Growth Studies and the WHO Fetal Study). Two of the three studies which established standards (the INTERGROWTH 21st Project and the WHO Fetal Study were done in multiple countries whilst the NICHD Fetal Growth Study was done only in the USA. The other 17 studies were done in single countries: four in Europe, four in Asia, three in North America, four in South America, one in the Middle East and one in North Africa. The purpose of the study by Dias et al. [18] was not clear and the remaining 16 studies produced foetal reference charts. Four of the 16 studies, which established references, were also longitudinal studies [11, 15••, 20, 22] whilst the remaining 12 studies were all based on a cross-sectional design.

Five studies established references for twins and 11 studies included healthy populations in their samples. The definition of healthy populations varied greatly amongst these studies making comparisons difficult. For example, Dias et al. defined healthy pregnant women as those who did not have a relevant past medical history; were not on long term medications; had no evidence of socio-economic constraints likely to impede foetal growth; no use of tobacco, recreational drugs or alcohol use; no evidence of urinary tract infections or renal disease on urinalysis; had a systolic blood pressure < 140 mmHg and diastolic blood pressure < 90 mmHg; no diagnosis or treatment for anaemia during pregnancy and not in an occupation with risk of exposure to chemicals or toxic substances [18]. Xu et al. included women without pre-pregnancy diabetes, pre-pregnancy hypertension, non-smokers during pregnancy, no alcohol consumption during pregnancy, self-reported pre-pregnancy body mass index (BMI) ≥ 17 kg/m2 or < 27 kg/m2, no pre-eclampsia and/or pregnancy-induced hypertension and non-diabetics [19]. Briceño et al. included women without maternal disease that may have affected foetal growth, such as hypertension, preeclampsia, diabetes mellitus and renal disease [3] which was similar to the inclusion criteria of Araujo Júnior et al. (women with absence of maternal diseases and absence of foetal malformations on sonography) [22]; Jiang et al. (women without maternal diseases, such as hypertension, pre-eclampsia, diabetes mellitus, renal disease; multiple pregnancies; and foetuses without congenital malformations, chromosomal abnormalities or intrauterine growth retardation) [27] and Kwon et al. (women without maternal disease possibly affecting foetal growth such as hypertension, diabetes mellitus and renal disease) [24], whilst Liao et al. included women with uncomplicated pregnancies [20]. Moety et al. also included only women who did not smoke and those without chronic diseases such as chronic hypertension or diabetes mellitus or give history of recurrent abortions [31].

Similarly, the three large prospective studies that developed foetal standards differed in their definition of what they considered as healthy women. In brief, the NICHD Fetal Growth Studies carried out by Buck Louis et al. excluded: women who smoked cigarettes or used illicit drugs in the past 6 or 12 months; who drunk ≥ 1 daily alcoholic drinks; had previous foetal congenital malformation; a history of non-communicable diseases (asthma requiring weekly medication, autoimmune disorders, cancer, diabetes mellitus, epilepsy or seizures requiring medication, hematologic disorders, hypertension, psychiatric disorders, renal disease and thyroid disease) or history of gravid diseases (gestational diabetes, severe preeclampsia/eclampsia or haemolysis, elevated liver enzymes and low platelet count syndrome) [15••], whilst the WHO Fetal Growth Studies included women with no socioeconomic constraints, normal daily caloric intakes and normal BMI [32••]. For the Intergrowth 21st Project, women were selected from urban areas located at low altitudes (< 1600 m). These areas were free from contaminants such as pollution, domestic smoke, radiation and other toxic substances. The definition of a healthy population in this study was: no clinically relevant past medical history, no history of sexually transmitted diseases, no history of a previous pregnancy affected by pre-eclampsia/eclampsia, HELLP syndrome or a related pregnancy-associated condition, no clinically significant atypical red cell alloantibodies, negative urinalysis, systolic blood pressure < 140 mmHg and diastolic blood pressure < 90 mmHg and commenced antenatal care before 14 weeks of gestation. Optimal nutritional status defined as first trimester maternal height (≥ 153 cm), body mass index (BMI, ≥ 18.5 and < 30 kg/m2) and haemoglobin concentration (≥ 110 g/L) without receiving supplements or long-term medications [5••].

We discuss some of the current issues in the development of foetal growth reference charts and standards in turn as informed by results of the scoping review of studies constructing foetal growth charts since 2012. These issues are by no means exhaustive but we believe represent some of key issues attracting debate in this field.

### How Charts Are Constructed—Prescriptive Vs Descriptive Approaches

One of the key differences in foetal growth charts is whether they are designed to be prescriptive or descriptive. Prescriptive charts describe the process of producing biological norms or a desirable target to be achieved or aspired to at individual and population levels (so as to construct growth standards). Prescriptive standards show how growth should occur, independent of time and place [33]. Prescriptive foetal standards refer to tools developed after carefully sampling healthy populations which have a low probability of foetal growth restrictions or abnormalities thereby limiting the effects of nutritional and environmental influences on growth patterns [4, 34]. The emphasis of these standards is to characterise optimal foetal growth and how foetuses should grow in the absence of factors known to affect foetal growth. For human growth, these are usually based on selected populations considered to be of optimal health (e.g., adequate nutritional status and at low risk of abnormal growth) for example the WHO Multi-centre Growth Reference Study (MGRS) [35••]. Until recently, it was generally accepted that observed differences in foetal growth were largely due to biological differences between different regions and ethnicities, resulting in a need for population-specific charts. This concept has been challenged by evidence demonstrating similarities in the genetic makeup of different non-isolated populations worldwide [36, 37] and more specifically by recent comparisons finding similarities in early and late linear foetal growth [38••] in diverse populations.

In contrast, descriptive charts are commonly used to produce a reference chart that describes the anthropometry of a given population at a particular time and place, such as a hospital, region or country. Descriptive reference charts are usually based on an unselected population with minimal exclusion criteria, for example, known risk factors for optimal health. Although they are used more widely, descriptive charts are only relevant to the source population. Different populations will differ in many aspects, such as rates of smoking during pregnancy, malaria, gestational diabetes and maternal obesity, which can all affect newborn outcomes. In principle, following the descriptive approach requires separate reference charts for each sub-population of interest.

Many descriptive charts are constructed from foetal measurements as a function of gestational age (GA) of the specified population. An alternative type of descriptive chart is the customised chart. Customised growth charts are constructed following a multivariable analysis that accounts for maternal factors known to affect foetal growth such as age, weight, height, BMI, parity, ethnicity, sex of the foetus etc. or paternal factors such as height [39]. An example of such a chart is the gestation-related optimal weight curve (GROW) chart by Gardosi et al. [39, 40••]. Unlike other foetal growth charts, the development of the customised charts does not need to exclude women based on their demographics as they are intended to be individualised and specific for each pregnant woman. The original computer-generated chart used data from 4179 births from a single hospital between 1989 and 1990 [40••] by first obtaining the 10th, 50th and 90th centiles at 40 weeks gestation and using a mathematical model to determine expected centiles at earlier gestations. The GROW chart was first constructed based on a UK population [40••], and the charts have been used in multiple populations including; Australia [41], the USA [42] and New Zealand [43].

There is still a debate on whether a unified international standard can be applied universally irrespective of location and ethnicity as demonstrated by three large prospective foetal growth studies published between 2014 and 2017 [5••, 15••, 32••].

We summarise the three studies’ key characteristics and features:

1. The INTERGROWTH-21st Project was based on a prescriptive approach with an aim to construct a single foetal growth standard for each foetal biometry measurement for international use despite ethnic differences based on overwhelming evidence from the WHO-MGRS that growth amongst healthy populations in diverse geographical settings is similar [35••]. The INTERGROWTH-21st Project was a longitudinal study conducted across eight different geographical settings; Brazil (Pelotas), China (Beijing), India (Nagpur), Kenya (Nairobi), Oman (Muscat), UK (Oxford), USA (Seattle) and Italy (Turin) in healthy populations demonstrated to have minimal constraints on foetal growth. The participant selection involved defining free-living populations in defined geographic areas with evidence of adequate health outcomes in terms of maternal, perinatal and neonatal morbidity and mortality and then selecting healthy pregnant women with good nutritional statuses and low risk of pregnancy complications from the well-defined populations [44]. The INTERGROWTH-21st project recruited over 4000 women prospectively for the construction of foetal standards. The study was conducted prospectively with recruitment carried out in the first trimester of pregnancy to ensure correct dating of the pregnancy. Follow-up antenatal ultrasound scans were performed every 5 weeks (± 1 week) by trained staff with identical ultrasound machines measuring both skeletal (head circumference, biparietal diameter, femur length and occipitofrontal diameter) and fat-based (abdominal circumference) growth measurement.
2. The WHO Fetal Growth Study is the foetal component of the WHO Multicentre Growth Reference Study, which aimed to establish growth charts for clinical use based on populations recruited from multiple countries—a similar aim to INTERGROWTH-21st Project [32••]. The WHO Fetal Study was a longitudinal prospective study of 1439 women recruited from ten countries i.e., Argentina, Brazil, Democratic Republic of the Congo, Denmark, Egypt, France, Germany, India, Norway and Thailand [32••]. Similar to the INTERGROWTH-21st Project, the study was done prospectively with recruitment of women in the first trimester between 8 and 13 weeks, who had reliable information on their last menstrual period confirmed by an ultrasound scan of the crown–rump length. The women were then scheduled for follow-up ultrasound scans which were performed monthly. The WHO Fetal Study also measured both skeletal and fat-based growth measurement of: head circumference, estimated foetal weight, both femur and humerus length, abdominal circumference and biparietal diameter. The WHO Fetal Study focussed on the estimated foetal weight charts to evaluate variation due to country, maternal characteristics (age, height, weight, BMI and parity) and sex of the foetus.
3. The National Institute of Child Health and Human Development (NICHD) Fetal Growth Study aimed to produce race/ethnic-specific foetal growth standards [15••]. This contradicts the prescriptive concept that one standard fits all. The study was, however, restricted to four self-reported ethnic groups of Asian, Hispanic, Black and White women in the USA. The study though prospective in nature, was hospital-based, with women recruited from 12 centres within the USA, who did not have any constraints on foetal growth or development. In total, 2334 women were recruited onto the study, with analysis performed on 1737 pregnancies. The women were recruited prospectively in the first trimester confirmed by a dating scan and were divided into the aforementioned ethnic groups. The women were then allocated into an ultrasound schedule that was designed to capture weekly foetal growth assessment data without subjecting all the women to weekly ultrasound scanning. As such, each woman attended five follow-up appointments. Similarly, both skeletal and fat-based measurements were undertaken; crown-rump length, head circumference, biparietal diameter, abdominal circumference and both the femur and humerus length until delivery.

### Attained Size Versus Growth—Utility of Foetal Growth Charts

There is a subtle difference between the growth of a foetus and the size of a foetus. In principle, size relates to measurements at a specific time, whereas growth relates to a change in size over time. Whilst foetal growth is evaluated from longitudinal measurements i.e., a series of anthropometric measurements made of each foetus at multiple time points [45, 46•, 47•, 48•], foetal size is determined at a single time point [33, 49, 50]. However, the term foetal growth is often used to describe both of these measurements and is thus sometimes used inappropriately [49, 51] as foetuses which are determined to have abnormal growth may actually be normal in attained size [45]. Longitudinal studies can therefore be used to produce both attained size and growth charts which have different interpretations and clinical applications.

### Population-Based Sampling Versus Hospital-Based Sampling

The choice of an appropriate sample and target population is of great importance as comparisons and inferences applicable to the general population can be made. The methodology of some of the studies used to develop foetal growth references sampled pregnant women from selected hospitals as opposed to sampling women directly from the population under investigation. The target population from which the women are selected has implications on whether the aim is to develop a reference or standard chart, generalisability and utility of the charts. For example, a chart based on women who are underweight cannot be applied to the general population. Hospital-based sampling could be problematic especially when there are varying levels of health services available to the population and when healthcare is provided by more than one health system service as is the case with several countries [34]. This could also be a potential source of bias in several low-income countries were a substantial number of women do not visit hospitals for pregnancy-related monitoring, prenatal and postnatal care.

### Period of Inclusion—Pregnancy Dating

The period of inclusion of the women into the study is also an important methodological consideration. For example, during the first trimester of pregnancy, there is less variability in foetal growth. Women recruited during this period using the first day of the last menstrual period could have this information confirmed using ultrasonographical evidence by measurement of the crown-rump length as it has been shown to be most reliable between 9^+0^ to 13^+6^ weeks gestation, but not beyond this range [52] and considered an essential part of routine antenatal care. Recruitment after the first trimester, leads to difficulties in ascertaining of accurate dating for estimating the expected date of delivery. A reliable estimate of gestational age is key as it underpins clinical care and allows the expected delivery date to be estimated accurately and also necessary for developing reference charts. Newborn outcomes such as preterm birth, small-for-GA, large-for-GA and appropriate-for-GA are all dependent on having an accurate estimate of GA.

### Study Design—Longitudinal, Cross-sectional and Mixed Designs

There are many design challenges for studies that aim to construct growth charts from foetal measurements. Foetal growth charts are designed to either monitor the foetal growth throughout the pregnancy to allow for medical intervention if required, or to determine the size of a foetus at a specified gestational age. Whether a study is longitudinal or cross-sectional in design is dependent on the question that it is trying to answer. Study design is of fundamental importance for any research study as it determines the appropriateness of the study to address the research question, and it helps inform the appropriate analysis of the data obtained. Most studies are based on a cross-sectional design [11] that include only one examination per foetus whereas a longitudinal design includes measurements at more than one time [48•]. It is common to construct size charts from longitudinal data by simply treating them as cross-sectional, as was done for example in the WHO Multicentre Growth Reference Study **(**MGRS) [35••]. The simplest case is a pure cross-sectional design, for example, Chitty et al. took one measurement per foetus at a random time [53]. A longitudinal design based on non-replicated data at each time point, ought to address correlated measurements from the same individual.

In contrast, a mixed design incorporates both longitudinal and cross-sectional measurements i.e., some participants are studied longitudinally and others cross-sectionally therefore for any given participant, the number of measurements included may be one or greater. A mixed design can be useful for studying growth intensively in periods of rapid growth using a longitudinal design and less intensively in periods of slow growth using a cross-sectional design. This may be an efficient, cost-effective approach especially for multicentre studies. An example is the WHO Multicentre Growth Reference Study (MGRS), which combined a longitudinal study design from birth to 24 months with a cross-sectional study of children aged 18 to 71 months [54]. A mixed design is also likely to arise when using routine data collected from individuals requiring close monitoring who are seen more than once.

### Statistical Considerations

Appropriate statistical methodology is key to the construction of foetal growth references and standards. A desirable feature of foetal charts is that centiles change smoothly with GA, and that the selected statistical methodology for fitting centiles provide a good fit to the raw data [46•, 47•, 55]. Some of the key statistical considerations include: (a) an assessment of whether the normality assumption is reasonable, as is usually the case for foetal data conditional on GA; (b) accounting for the increasing variability with gestation that is typical in foetal growth data; and (c) a goodness-of-fit assessment with graphical evaluation of the superimposed centiles should be conducted to compare the predictive model to the raw data.

### Sample Size

There is very limited literature on what to consider when determining the sample size of foetal growth studies [56–59]. A systematic review of the methodology used in published ultrasound studies for developing size or pregnancy dating charts found that only 6 of 83 published ultrasound growth or size charts included their sample size calculations in the description of their methodology [11, 60].

Sample size calculations can be based on either parametric or non-parametric methods. Non-parametric methods can be implemented using simulation and bootstrap techniques as has been demonstrated by Harris et al. [61], Linnet [62] and Jennen-Steinmetz [58]. Regression-based methods for sample size can also be evaluated by either non-parametric or parametric approaches depending on the distribution of the covariate [63, 64]. For example, methods based on regression-based limits are commonly used in clinical chemistry studies involving normal reference ranges [65]. These methods can be adopted and applied in foetal and neonatal growth studies [66]. Formulae for estimating sample size for regression-based reference ranges were first proposed by Royston [57] and later extended by Bellera and Hanley [56]. In 2011, Hanley and Moodie [67] proposed a unified approach for sample size, precision and power calculations that considers various study designs. Later in 2016, Hanley [59] discusses sample size considerations for the case of simple and multiple linear regressions. Regression analysis can be used to obtain reference limits that account for factors such as age, gender and parity with corresponding confidence intervals (CIs) [68–71].

Precision and power are the key factors in the determination of sample size for constructing reference charts in addition to study design (longitudinal, cross-sectional or mixed), number of repeated measurements per individual, existence of replicate measurements and practicality (cost, time and manpower) [72]. The precision of estimated centiles is inherently variable. Extreme centiles exhibit large imprecision because there are few observations at the extreme ends of the distribution, whilst the median has the greatest precision. For normally distributed unreplicated data, the standard error of the *p*^th^ centile is obtained from the standard formula for the variance of a centile [73]:

$$ \mathrm{SEp}=\mathrm{SD}\surd \left[\left(1+\frac{1}{2}{z}_p^2\right)/n\right], $$

where SE is the standard error, SD is the standard deviation of the measurement (which will increase with GA), z_p_ is the value of the standard normal distribution corresponding to the *p*^th^ centile, and *n* is the sample size. For example, for the 2.5th or 97.5th centiles, zp = ±1.96, giving SE = 0.08 SD with a sample size of 500 and 0.03 SD for a sample of 4000. More extreme centiles will require a larger sample size to estimate than less extreme ones for the same precision. It is also advisable and common practice to inflate the calculated sample size by the expected percentage of attrition for the specific setting.

In general, longitudinal studies are more efficient and have greater power than cross-sectional studies. Royston (1995) defined this efficiency as the design factor, D, which is the number of foetuses in a cross-sectional study that would give the same precision as one foetus in a longitudinal study. He used a simulation study of ultrasound-based biparietal diameter and compared the variance of a centile in longitudinal and equivalent cross-sectional designs. He calculated the design factor (effect) to be ~ 2.3 [74]. A longitudinal study thus requires approximately half to a third the sample size of a cross-sectional study to estimate a given centile with the same precision depending on the number of measurements per foetus. In the case of subgroups or multicentre studies, a sufficient power may be required in order to explore ethnic-specific (i.e., site-specific) charts.

### Handling Data from Multiple Sites

Most studies aiming to construct foetal growth references are done in a single centre. The need for a large sample size and greater generalisability leads naturally to a multicentre design, which brings additional challenges. As multicentre studies are rare in human growth studies, the combinability problem is not common. However, the MGRS, INTERGROWTH-21st Project, NICHD and WHO Fetal Study were multicentre studies and so faced this problem. Statistical significance is not appropriate for judging combinability, as even unimportant differences can be statistically significant especially in very large samples. Assessing how appropriate it is to pool data from multiple sites is challenging, as a judgement of the similarities in the foetal growth size patterns across the populations must be made. The combinability of studies in a meta-analysis is usually judged qualitatively using the similarity of the studies, such as the similarity of the participants, interventions and outcome variables. This is akin to the standardised and careful selection process employed by the studies which strives to ensure similarity of women selected from different sites. Judgments on similarity of data from different sites depends on quantifying the differences and variability inherent in the data for which there is no standard statistical approach for evaluating what is an acceptable level of agreement.

Some considerations on how to make judgments on similarity of data include: defining a priori threshold of acceptable differences based on clinical knowledge for judging whether the differences between the centile curves from each site are acceptable before conducting the analysis, conducting a sensitivity analysis of the inclusion/exclusion of specific data to the overall fitted centiles, quantifying the amount of variability that can be attributed to site differences and defining a priori on what differences are considered acceptable based on clinical impact or meaningfulness.

For example, the INTERGROWTH-21st Project used the same criterion as the WHO-MGRS where the impact of the consistency and magnitude of differences in each site compared to all sites was judged according to Cohen [75] with differences of 0.5 SD considered to be medium (an ideological criterion rather than a statistical criterion). This criterion is also widely used in the assessment and evaluation of changes in health-related quality of life measures and patient reported outcomes [76]. Therefore a difference of 0.5 SD or greater (defined a priori) between the centile curves from a site and the combined data from all of the sites at any GA would indicate that the data from that site were too different to be pooled [55, 77•]. In addition, a sensitivity analyses involving an assessment of the impact of excluding each site’s data one at a time on the overall fitted centiles derived from all the pooled data is useful in making judgments on whether any single sites data is incompatible with the rest of the data from other sites or countries. It is recommended that multicentre studies should quantify and evaluate the differences between their sites using pre-specified criteria, as was done in the INTERGROWTH-21st Project and the WHO MGRS study [77•].

## Concluding Remarks

In this paper, we have provided a scoping review of studies constructing charts between 2012 and 2018. The review clearly demonstrates a lack of consensus on how foetal charts should be constructed and whether an international chart that can be applied across populations is feasible. We have discussed some of the pertinent issues emanating from the review, discussed current developments and debates in the construction of foetal references and standards. We have highlighted some issues regarding how foetal growth reference charts are constructed (prescriptive and descriptive approaches), and the study design and methodological considerations for constructing reference centile charts. Important design features such as inclusion and exclusion criteria, sample size determination, gestational age (GA) estimation and handling of data from multiple sites for multicentre studies are seldom well addressed, considered or reported.

As many of these charts are in clinical use today and directly affect the identification of at risk newborns that require treatment and nutritional strategies, the establishment of foetal biometric charts for use require careful methodological considerations. The observations by David Barker in the late 1980s, on the association between early growth parameters such as birth weight and the risk of disease in later life [78, 79] leading to the famous ‘Barker’s hypothesis’ reiterates how crucial and important the first 1000 days of life is. They confirmed the already-overwhelming evidence that foetal growth disorders are risk factors for adverse perinatal outcomes and can predispose infants to adult chronic diseases [80–84]. These findings on early foetal programming and associated risk of disease in adulthood stimulated lots of interest amongst researchers culminating into the formation of an international society for developmental origins of health and disease (DOHaD). This is particularly important as there is still an ongoing debate on whether a single growth standard chart can be used internationally [4]. Those who argue against this suggest significant differences between racial/ethnic constructs sufficient enough for the production of racial/ethnic-specific charts for use in foetal growth monitoring implying a significant influence of a genetic component in foetal growth patterns existing across ethnicities. Proponents for a single growth standard for international use argue that differences observed in foetal growth patterns arise mainly due to socioeconomic factors like nutritional status and environmental exposures [34].

As demonstrated in Table 2, there have been numerous charts developed for both local (e.g., Liao et al. 2012 [20]), international (e.g., Papageorghiou et al. [5••] and Kiserud et al. [32••]), as well as customised use [85]. The current discussion around foetal growth charts is whether ethnicity plays a role in foetal growth and therefore whether it should be taken into consideration in the creation of the charts. A comparison of the INTERGROWTH-21st Project, NICHD and the WHO-sponsored foetal charts found that there were minimal differences between the three charts in terms of head circumference across gestational ages [34]. The INTERGROWTH-21st Project constructed charts from eight diverse populations following similar methodology, recruitment and standardisation and demonstrated that there was great similarity in foetal growth amongst healthy women who were well nourished and lived in good environments. The NICHD study hypothesised that there are differences in foetal growth by ethnicity and therefore aimed to construct ethnic specific charts. Gardosi et al. have always argued for the need of customised charts that account for a woman’s characteristics that are known to affect growth such as height, weight and BMI, and have constructed charts that include these variables.

In summary, we have highlighted some of the current issues related to the development of foetal references and standards. The systematic review of foetal charts published in the last 5 years shows that these issues still recur with different opinions on how these charts should (or should not) be constructed. There is no consensus on these pertinent issues and hence if not resolved will lead to continued surge of foetal reference and standard charts which will only exacerbate the current problem of not being able to make direct comparisons of foetal size and growth across populations.
